# The Future of Graphene: Preparation from Biomass Waste and Sports Applications

**DOI:** 10.3390/molecules29081825

**Published:** 2024-04-17

**Authors:** Yueting Wu, Yanlong Li, Xiangyang Zhang

**Affiliations:** 1 Graduate School, Harbin Sport University, Harbin 150008, China; wuyueting@hrbipe.edu.cn (Y.W.);; 2 Academic Theory Research Department, Harbin Sport University, Harbin 150008, China

**Keywords:** graphene, biomass waste, sports equipment, graphene derivatives, biocomposites

## Abstract

At present, the main raw material for producing graphene is graphite ore. However, researchers actively seek alternative resources due to their high cost and environmental problems. Biomass waste has attracted much attention due to its carbon-rich structure and renewability, emerging as a potential raw material for graphene production to be used in sports equipment. However, further progress is required on the quality of graphene produced from waste biomass. This paper, therefore, summarizes the properties, structures, and production processes of graphene and its derivatives, as well as the inherent advantages of biomass waste-derived graphene. Finally, this paper reviews graphene’s importance and application prospects in sports since this wonder material has made sports equipment available with high-strength and lightweight quality. Moreover, its outstanding thermal and electrical conductivity is exploited to prepare wearable sensors to collect more accurate sports data, thus helping to improve athletes’ training levels and competitive performance. Although the large-scale production of biomass waste-derived graphene has yet to be realized, it is expected that its application will expand to various other fields due to the associated low cost and environmental friendliness of the preparation technique.

## 1. Introduction

In recent years, with the continuous advancement of a new round of global scientific and technological revolution and industrial upgradation, the requirements for new materials in various fields have become more stringent and diversified. The traditional crystals are too unstable to exist as two-dimensional (2D) structures at non-zero absolute temperatures, which has long plagued researchers. However, the discovery of graphene has successfully filled this gap and completed the graphitic materials system. Graphene—a nanomaterial—consists of a single layer of sp^2^-hybridized carbon atoms arranged in two dimensions with a hexagonal honeycomb crystal structure. So far, graphene is the thinnest nanomaterial known and is considered the “magic material of the 21st century”. It possesses many excellent properties, such as a large surface area, high conductivity, good chemical stability, and excellent mechanical strength [1,2,3,4], which are vital for applications spanning electronics, aerospace, renewable energy systems, sports equipment, and other technologies. In addition, the emergence of this material has spawned a series of new research fields targeted at future needs.

**Figure 1:**
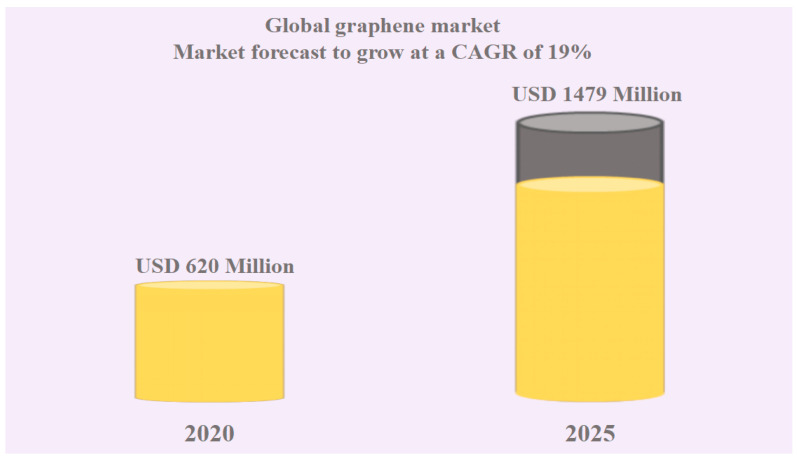
Estimated CAGR of the Global Graphene Market (adapted from Ref. [5]).

Presently, the proportion of downstream application enterprises of graphene is gradually increasing, and its market is slowly unfolding. Figure 1 shows a new wave of upsurge in the graphene market, which is expected to reach USD 147.9 billion by 2025 [5]. Currently, the primary precursor materials for graphene are mainly metal-based, coal-based, or petroleum coke-based [6,7,8,9,10]. However, these materials are associated with many problems, such as soil and water pollution, releasing harmful gases and particulate matter, and atmospheric pollution, significantly limiting their application [11].

The biomass waste generated in the world is increasing year by year, most of which is buried and incinerated [12,13]. This type of treatment not only causes the waste of resources but also leads to environmental pollution. To overcome the challenges associated with the traditional methods of graphene production, such as environmental problems, resource scarcity, and high costs, the research on graphene production from biomass materials has attracted much attention in recent years. Scholars actively seek greener, cleaner, and more efficient materials, including different biomass materials, such as biomass waste resources (composed of plants and animal waste) [14] in agriculture, forestry, and municipal solid waste (MSW), to reduce the economic cost and environmental impact of graphene production. This report reviews the latest progress in the research and development of graphene prepared from biomass waste. The basic structure, characteristics, derivatives, and production methods of graphene are summarized with a detailed discussion of the inherent advantages of selecting biomass waste as a precursor to its preparation. Although many relevant studies on graphene have already been conducted, a detailed focus on its application in sports equipment is still lacking in the literature. Therefore, this report fills the literature gap by focusing on the application prospects of graphene and its derivatives in the sports industry. Finally, the challenges and future outlook of biomass waste-derived graphene are presented from the perspective of the sports industry.

## 2. Graphene Overview

Graphene is a 2D single-atomic-layer material with a honeycomb lattice structure of closely arranged carbon atoms. Due to its thermodynamic instability, graphene has been used as a theoretical model to describe the structure of other carbon materials before it was discovered [1]. The relevant academic research on graphene can be traced back to 1947 when Wallace et al. [2] proposed a theoretical model of graphene. This breakthrough model established the starting point of academic research on graphite electrical properties. In 2004, physicists Novoselov et al. [15] of the University of Manchester in the United Kingdom practically obtained single-layer graphene for the first time using a tape-stripping method. This breakthrough attracted much attention and inspired scholars to conduct in-depth research on graphene’s structure, properties, and derivatives.

### 2.1. Graphene Structure

**Figure 2:**
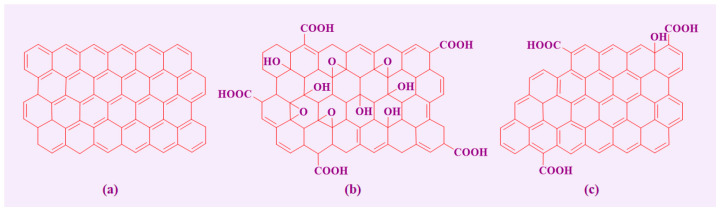
The structure of (**a**) graphene honeycomb, (**b**) graphene oxide (GO), and (**c**) reduced graphene oxide (RGO) (adapted from Ref. [21]).

Figure 2 shows the structure of graphene and its derivatives, which exhibit a honeycomb-like shape with individual carbon atoms evenly distributed in a 2D lattice, each having 2 s and 2 p electrons in its outer shell [16,17]. The s and p orbitals are sp^2^-hybridized to make a typical hexagonal benzene ring structure, which provides the material with significant mechanical stability [18]. In addition, there are p_z_ orbitals perpendicular to the direction of the plane, forming π chemical bonds. The electrons involved in these π bonds are crucial for the conductivity of graphene, which exhibits different electronic band structures depending upon the structure of the edge carbon chain and the number of staked layers in graphene [19,20].

Graphene has a mechanically stable chemical structure of short C-C bonds (0.142 nm length) connected in a solid fashion [22]. Consequently, when an external force is applied to the sheet, the bond lengths deform to offset the external stress without rearranging the individual carbon atoms, thus maintaining a stable structure [23]. Moreover, scanning tunneling microscopy (STM) shows that the surface of graphene possesses some nanoscale wrinkles [17], which helps overcome the limitations of thermodynamic fluctuations, enabling the stable existence of the 2D lattice [24].

### 2.2. Graphene Properties

Carbon has achieved a quantum leap from graphite to graphene with a fully exposed bulk and edge atomic arrangement on its surface, which endows it with excellent mechanical, electrical, optical, and other properties. Researchers have conducted in-depth studies on graphene properties and have continuously identified surprising discoveries, which have aroused the interest of many interdisciplinary scholars.

Graphene exhibits high mechanical strength, good toughness, and excellent flexibility. The strong σ and π covalent bonds formed between the graphene carbon atoms are mainly responsible for the superior mechanical properties and structural rigidity [25]. Since graphene is extremely thin in atomic order, its thickness is challenging to measure directly through experimentations. Consequently, determining Young’s modulus of graphene is more challenging than that of bulk materials. Lee et al. [26,27] pioneered the study of graphene monolayers’ elastic properties and inherent strength using nanoindentation technology based on atomic force microscopy (AFM). The results showed that Young’s modulus of graphene monolayers was as high as 1.1 TPa, and its breaking strength could reach up to 42 N m^−1^. Interestingly, graphite paper composed of graphene exhibited weak toughness initially, but its toughness was significantly enhanced following oxidation and functionalization. In addition, Lee et al. [28] demonstrated graphene balloon technology by preparing graphene through chemical vapor deposition (CVD), transferring it to a circular hole, and placing the sample in a vacuum. Due to the airtightness of graphene, air could not penetrate it, resulting in a higher air pressure inside the balloon. The researchers used Raman spectroscopy to study the balloon and compared the results with numerical simulations. The study concluded that Young’s modulus of monolayer graphene was about 2.4 ± 0.4 TPa.

Graphene is very sensitive to its environment due to its unique 2D structure. Its interactions with other materials may affect its electrical and optical properties and trigger new physical phenomena. Most applications based on graphene rely on its high electronic mobility. Electrons in graphene are free to move owing to the extended delocalized bonds. However, the mobility is hindered by internal structural defects and scattering due to lattice vibrations [29], which are almost independent of temperature [30]. Therefore, at room temperature, when the carrier concentration is about 10^12^ cm^−2^, due to scattering by phonons, the charge mobility can be as high as 200,000 cm^2^V^−1^s^−1^, which is almost 140 times that of commercial crystalline silicon [31]. Moreover, graphene has excellent optical properties, especially high optical transmittance. When white light is irradiated vertically on single-layer graphene, only about 2.3% of the light is absorbed, resulting in a transmittance of 97.7% [32]. As the number of layers increases, the visible light transmittance decreases by 2.3% for each successive layer. Non-interactive Dirac–Fermi simulations showed that the transmittance in graphene was affected by its thickness, and the number of graphene layers could be estimated from the value of transmittance [33,34]. In addition, the optical transitions in graphene could be modulated by changing its Fermi level with an applied gate voltage. For instance, when graphene absorbs photons, the generated electron-hole pairs can recombine in a very short time of picosecond. However, when an external electric field is applied, the excited electron-hole pairs are separated, resulting in a photocurrent flow [35,36,37]. Due to the unique zero-bandgap structure of graphene, the energy required for electrons to transition from the valence band to the conduction band is almost zero (theoretically calculated). Consequently, the majority spectrum of incident light can generate a certain photocurrent intensity in graphene. Furthermore, graphene can also be excited by appropriately modifying its zero bandgap [38].

### 2.3. Graphene Derivatives

Graphene derivatives are chemical modifications based on graphene, imparting novel properties to the material, thus significantly expanding its application fields [39,40]. Table 1 compares the mechanical properties of the graphene family.

Graphene oxide (GO) is the prime representative of graphene-derived materials, also known as graphite oxide. It is prepared by the chemical oxidation of graphite powder. Due to variations in the oxidation conditions, the structure of GO has not been understood in detail until recently. As per the most widely accepted model of GO, the most active functional groups, such as epoxy (-O-) and hydroxyl (-OH), are mainly distributed on its surface. In contrast, functional groups, such as carboxyl (-COOH), carbonyl (-C=O), and phenol (Ar-OH), are connected to the edges of the GO sheet [43]. Moreover, the number of functional groups on GO depends on the preparation method employed. 

GO exhibits properties utterly different from those of graphene due to its many oxygen functional groups and its layer structure, which markedly differs from the latter. Compared with graphene, GO exhibits stronger hydrophilicity because of its ability to make hydrogen and polar bonds with water molecules. This property makes GO easy to exfoliate and disperse stably in water and suitable organic solvents [44,45]. However, compared with graphene, oxygen-containing functional groups can lead to structural defects in the 2D atomic structure of GO, resulting in a corresponding loss of mechanical and electrical properties [46,47]. 

GO can easily disperse in water to make a stable solution that can facilitate its deposition on suitable substrates through the standard deposition techniques of drop casting, spraying, and spin coating [17]. At the same time, these oxygen functional groups can facilitate the covalent or non-covalent binding of the material to other substances, further expanding GO applications [48]. However, the large number of oxygen-containing functional groups on the surface of GO disrupts the continuous conjugated structure of graphene, resulting in the loss of the characteristic high thermal and electrical conductivity of the material. To re-establish the conjugated structure and unique properties of graphene, researchers chemically reduce GO to obtain reduced graphene oxide (rGO) with partial recovery of the conjugated structure associated with unavoidable defects.

rGO is, therefore, also a graphene derivative, which has properties similar to graphene, particularly excellent conductivity and mechanical properties [49]. The GO reduction process removes the oxygen-containing groups and restores the conjugated structure of graphene. Different reduction methods (including thermal, chemical, microwave, photochemical, and hydrothermal reduction) lead to significantly different quality of the final rGO. Chemical and thermal reductions are the two most widely used methods. Chemical reduction usually employs chemical reducing agents, including hydrazine, ascorbic acid, hydroiodic acid, and sodium borohydride, to treat GO in rGO. Among the various reducing agents, ascorbic acid has gained particular interest for the scalable production of rGO without generating toxic gases [50]. Thermal reduction, on the other hand, employs high-temperature conditions to remove oxygen-containing functional groups on the GO surface. Theoretically, GO can be completely deoxidized under extreme temperatures of more than 2000 °C. 

Following the elimination of the oxygen-containing groups, the rGO shows completely different properties to GO in some respects [51]. For instance, rGO exhibits better chemical stability and electronic conductivity than GO. Moreover, rGO is prone to aggregation in aqueous and related media, thus controlling its shape and specific surface area [52]. Conversely, oxygen-containing groups significantly enhance the hydrophilicity of GO, making it more stable to disperse uniformly in water [53]. These characteristics open up new opportunities for rGO and rGO-based devices in various fields.

Three-dimensional graphene (3D graphene) is a derivative of graphene consisting of longitudinally grown stacks of 2D graphene, preserving many of its inherent properties, such as high electrical conductivity. In addition, it possesses unique structural/electrical characteristics, resulting in the increased absorption of near-infrared (NIR) to infrared (IR) radiations by up to 16% [54]. The other most common chemical derivative of graphene is nitrogen-doped graphene. Nitrogen is usually present in various carbon-based materials; however, its content is low, with no well-defined stoichiometry. The presence of heteroatoms in the aromatic carbon rings of graphene interferes with its aromaticity, resulting in various effects, including bandgap opening [55,56], charge separation, ferromagnetic ordering, different interlayer forces, and catalytic potential [57]. Subsequently, the properties of pristine graphene are altered, providing broad prospects for its application in specific fields, such as nanoelectronics, electrochemical biosensors, energy storage, and other fields. 

## 3. Preparation Method of Graphene

The preparation of graphene plays a crucial role in advancing its scientific research and wide applications. Different preparation methods vary in the yield and quality of the resulting graphene, which are suitable for different applications [58]. Achieving the large-scale, automated, low-cost production of high-quality graphene is essential for manufacturing high-performance graphene devices. The preparation methods reported in the literature can be classified into the following two categories: top-down and bottom-up approaches [59,60,61]. The top-down approach aims to break down bulk graphite into lamellar graphene sheets through exfoliation. Conversely, the bottom-up approach assembles graphene from individual carbon atoms using precursors such as methane or polymers with benzene rings and is advantageous for high-yield production. Table 2 compares the different preparation methods of graphene, including the typical micromechanical lift-off, CVD, redox synthesis, and epitaxial growth methods. 

### 3.1. Micromechanical Lift-Off

The mechanical exfoliation method was the earliest method used to prepare multilayer graphene. This process of graphite delamination was achieved by applying a mechanical force. Geim et al. [66,67] successfully separated graphene nanosheets by repeatedly sticking adhesive tape to highly oriented pyrolytic graphite (HOPG) to obtain layered graphene, combined with applying oxygen plasma etching. The principle of this preparation method is based on the fact that van der Waals forces between graphite sheets are weak and, thus, can easily be separated by applying an external force. Paton et al. [68] reported a simple and efficient method for producing graphene using liquid-phase exfoliation. Graphite powder was placed in an organic solvent containing a specific surfactant and subjected to high-energy shear using a stator-rotor homogenizer (made by Silverson Machine, East Longmeadow, MA, USA). This method effectively improved the speed of graphene production via mechanical exfoliation. Furthermore, the automated exfoliation method has significant advantages, including the simplicity, low cost, and high quality of the resulting graphene. However, the graphene prepared through this method is highly laborious and time-consuming. Additionally, there are uncontrollable factors causing difficulty controlling wrinkles and the number of layers. As a result, this method is limited to laboratory research and is challenging to meet the demands of scaled-up production [69].

### 3.2. Chemical Vapor Deposition

The CVD technology originated in the 1960s and was initially used for thin film preparation in the semiconductor industry. Later, it gradually expanded to the field of nanomaterials. In recent years, this technology has played an essential role in promoting the development of high-quality graphene. Before introducing the CVD technology to prepare graphene, researchers found that layered graphitic materials can be formed on nickel (Ni) substrates through high-vacuum annealing [70,71]. The CVD preparation is mainly divided into two types based on the growth mechanism: (1) the dissolution precipitation mechanism, employing Ni, cobalt (Co), and other metals with high carbon dissolution ability, and (2) the surface catalytic mechanism, utilizing metals with low dissolved carbon content, such as copper (Cu), molybdenum (Mo), and platinum (Pt). In the former mechanism, the dehydrogenation of the carbon source produces carbon atoms that penetrate the metal substrate at high temperatures. When rapidly cooled, the carbon atoms precipitate out of the interior of the metal to nucleate and grow into graphene on the surface of the substrate [72,73,74,75,76]. On the other hand, in the latter mechanism, the carbon source is dehydrogenated at high temperatures to generate activated carbon atoms, which, after reaching a certain supersaturation on the metal surface, leads to nucleation and growth to form the graphene crystal domains. Finally, continuous graphene is obtained through 2D growth and merging of the crystal domains precipitated on the surface of the substrate [77,78]. 

CVD technology can prepare single-crystal graphene films with sizes ranging from 10 microns to 1.5 inches [79,80]. Park et al. [81] used CVD technology to prepare few-layer graphene (FLG) on nickel-coated substrates and found that chemical bonds connected different crystal domains of the FLG. Although the FLG samples were not uniform in macrostructure, they demonstrated excellent mechanical properties. The advantages of CVD technology include the preparation of graphene with a large 2D area, superior quality, and complete lattice structure. However, this technology is challenged by the difficulty of separating graphene from the metal substrate and the possible product contamination of the organic molecules used for its separation.

### 3.3. Oxidation–Reduction Method

rGO is prepared by the oxidation–reduction method. Generally, graphite is first oxidized using a strong acid and oxidant and then exfoliated into GO by ultrasonic treatment. Finally, the oxygen-containing functional groups on the surface of the GO are reduced to obtain rGO. Various oxidation strategies, including Brodie, Hummer, and Staudenmaier methods, are commonly found in the literature [82,83,84]. Since the synthesized GO is rich in polar functional groups, it can be dispersed in water or alkaline solutions by ultrasonication. Then, GO flakes are reduced using various methods, such as microwave reduction, solvothermal reduction, vapor phase reduction, and chemical reduction [51,85,86]. It is noted that rGO prepared through the redox method destroys the π-electrons conjugated structure, resulting in more functional groups and other types of defects in the synthesized rGO, thus compromising its performance [87,88]. However, a large amount of only a few layers of rGO could be obtained by this method, offering a low-cost production [89,90,91,92] demanded by various fields of application.

### 3.4. Epitaxial Growth Method

In the early 1970s, graphene growth on a nickel single crystal (111) surface was studied [93]. A graphene-decorated SiC substrate was obtained by heating a silicon carbide (SiC) single crystal at high temperatures for the silicon atoms to evaporate, which is associated with the reconstruction of the carbon atoms through self-assembly. Initially, the growth conditions for graphene on a nickel single crystal (111) surface required an ultra-high vacuum (UHV). When a clean Ni (111) surface is exposed to hydrocarbons, surface carbide or graphene is produced. Additionally, it has been confirmed that the thermal decomposition of hydrocarbons at low temperatures leads to the formation of surface carbide, while graphene typically forms at temperatures between 500 °C and 700 °C [94]. However, recent studies showed that an atmosphere of argon or small amounts of disilanes can reduce the rate of silicon sublimation, allowing better-quality graphene to be prepared at higher temperatures [95]. Hass et al. achieved controlled single and multilayered graphene growth using the SiC decomposition method [96]; however, multilayer graphene up to 100 layers is relatively easy to obtain. The quality of graphene prepared through this method is closely related to the heating temperature, reaction pressure, and protective gas type. Therefore, high-quality graphene can be obtained by adjusting the reaction parameters using the epitaxial growth method. However, the graphene yield is low while the reaction temperature is high, limiting its adaptation for the large-scale synthesis of single-layered graphene [97,98].

## 4. Biomass Waste-Derived Graphene

**Figure 3:**
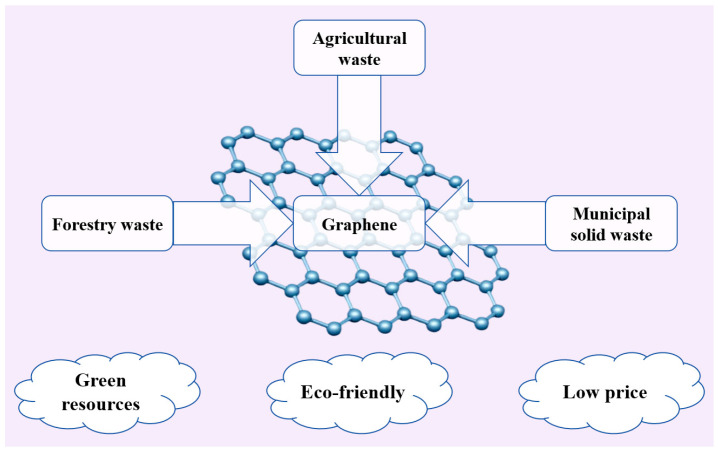
Raw materials and advantages of green synthesis of graphene (adapted from Ref. [102]).

With the increasing emphasis on resource sustainability and environmental protection, people are gradually becoming aware of the broad prospects of biomass resources [99,100]. Biomass waste, including biomass waste from agriculture, forestry, and MSW, constitutes an integral part of biomass resources, which is cheap and widely available [101]. Figure 3 shows the raw materials and advantages of green synthetic graphene.

**Figure 4:**
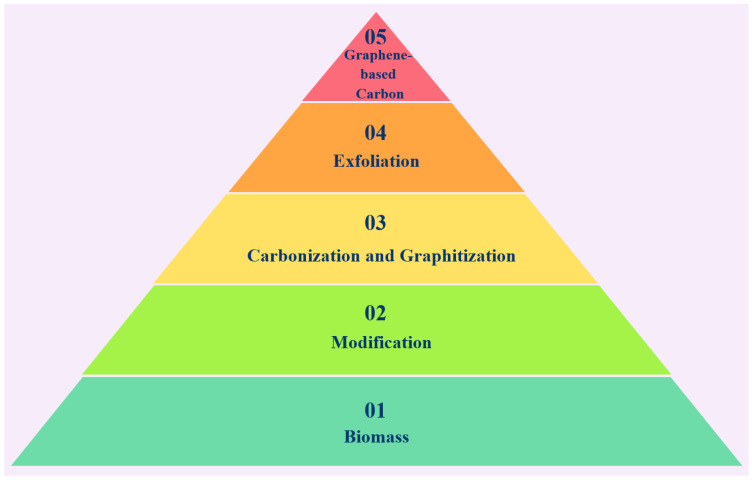
Strategies for using biomass to produce graphene. (adapted from Ref. [103]).

The treatment and recycling of biomass waste directly impact the comprehensive utilization and efficiency of natural resources. Following proper treatment and processing, biomass waste has the potential to produce high-value-added chemicals and carbon materials. Therefore, biomass waste recycling is expected to promote the synergistic development of environmental protection and resource utilization. Figure 4 shows various strategies for utilizing biomass to produce graphene.

### 4.1. Agricultural Waste

Globally, large quantities of agricultural products are produced daily to meet the needs of a growing population, leading to the large-scale incineration of agricultural waste [104,105,106]. The indiscriminate incineration of these wastes releases harmful substances, such as carbon monoxide, sulfur dioxide, and dust, which are significant sources of air pollution and pose a severe threat to respiratory health [107]. At the same time, the disposal of agricultural waste without proper treatment may also cause various hazards, such as the spread of infectious diseases, water pollution, and ecosystem damage. 

Agricultural waste mainly includes three organic substances: cellulose, hemicellulose, and lignin. Hemicellulose is a heteropolysaccharide whose specific composition varies according to its source. For instance, hemicelluloses from angiosperms and gymnosperms, composed of xylans and glucomannan, respectively [108], are excellent carbon sources for synthesizing carbon materials. In addition, agricultural waste provides abundant raw materials for producing high-performance graphene, with significant advantages in adsorption performance and the quality of the obtained graphene [109].

Rice is one of the major sources of food. The rice husk is the outer covering of the rice grains. During growth, rigid silica in the husk protects rice from insects and bacteria and ensures it can obtain the necessary water and nutrients. To achieve dual functions, silica in rice husks has evolved naturally into a unique 3D porous nanostructure [110,111,112]. Wang et al. [113] reported biocompatible graphene quantum dots derived from rice husks with a yield of 15% and obtained mesoporous silica nanoparticles as a by-product of the preparation, realizing the comprehensive and effective utilization of agri waste.

Wheat straw is the stalk left after wheat harvesting, and its main components include cellulose, hemicellulose, lignin, trace metals, and crude proteins [114]. Chen et al. [115] used potassium hydroxide (KOH) to dissolve hemicellulose and lignin in wheat straw. After multiple heat treatments to overcome its complex structure, wheat straw was finally converted into cellulose fibers, which, like other lignocellulosic materials, could be converted into graphene using high-temperature carbonization (HTC). Zhou et al. [116] successfully synthesized interconnected high-graphite carbon nanosheets (HGCNSs) using wheat straw as a precursor and combining the hydrothermal and graphitization processes. The experimental results showed that HGCNS possessed an interconnected two-dimensional nanostructure capable of providing multiple storage sites for lithium ions and facilitating the rapid transport of electrons and ions. Additionally, due to its high degree of graphitization, HGCNS significantly reduced voltage hysteresis, demonstrating excellent cycling and rate performance. Graphene prepared from agricultural waste has significant sustainability, cost-effectiveness, and environmental advantages, reducing resource waste and environmental pollution while simultaneously achieving multifunctional applications.

### 4.2. Forestry Waste

Forestry waste is a vital biomass resource. Energy conversion utilizing forestry waste is one of the research hotspots in renewable energy. Forestry waste, mainly including branches, sawdust, rolled bark, shavings, and scraps, is continuously generated in forestry processing. Whether woody plants or herbs, their cell walls are primarily composed of cellulose, hemicellulose, and lignin [117], indicating that their structure has a high carbon content. Eucalyptus bark extract is rich in 29 polyphenol compounds, which are considered bioactive due to their excellent antioxidant and anticancer properties. Manchala et al. [118] synthesized soluble graphene using a eucalyptus polyphenol solution obtained from the Eucalyptus bark extract. The results showed that the polyphenol compounds in Eucalyptus bark extract had a reducing ability; therefore, exfoliated GO could be reduced to soluble graphene under reflux in an aqueous medium.

Wood processing produces a tremendous quantity of waste wood. Although the wood-based panel processing industry consumes many wood processing residues, wood chips are more cumbersome. Therefore, these biomass resources have not been recycled and reused effectively as fuel. Although sawdust has not been widely used for fuels due to its chemical composition, it still has the potential to produce high-quality, high-value-added carbonaceous materials such as graphene. Severo et al. [119] successfully prepared 3D graphene sponges from sawdust by pre-carbonization and KOH chemical activation. In addition, this method achieves the high value-added utilization of sawdust, helping to reduce biomass waste generation and protect the environment. 

### 4.3. Municipal Solid Waste

MSW is a heterogeneous waste stream, which is an inevitable part of daily life [120], characterized by complex components and a high organic content. On average, 0.74 kg of waste is generated per person per day globally. The per capita waste generation is closely related to the local economic level and the degree of urbanization. Generally, the higher the income, the higher the per capita waste generation. Moreover, global waste generation is growing more than twice the population growth rate and is projected to reach 3.4 billion tons by 2050 [121].

Disposable paper cups (DPCs) are widely used in many families, enterprises, and public places due to their relative hygiene, low cost, convenient use, and mass production [122]. However, most DPCs have a short service life and are difficult to segregate from waste after use. They are eventually incinerated or buried in landfills, posing a severe threat to human ecosystems. DPCs usually comprise high-grade cardboard and an inner polyethene coating. Wang et al. [113] proposed a new route to prepare Fe/graphene sheets using Fe^2+^ catalysts with DPCs as a carbon source. It was demonstrated that the proposed synthesis strategy could produce graphene sheets with high yield and high quality along with two additional products, Fe/graphene and Pt/graphene, where the latter showed high catalytic activity for an oxygen reduction reaction in fuel cells. Ruan et al. [123] used inexpensive carbonaceous raw materials without pre-purification, including biscuits, chocolate, grass, plastic, cockroaches, and dog feces, to grow graphene directly on copper foil using CVD technology. The results showed high-quality graphene monolayers prepared from these carbon sources. Graphene production using MSW has potential in terms of quality; however, it currently faces challenges regarding yield. With process optimization, this method could become a competitive route to produce high-quality graphene.

## 5. Graphene Applications in Sports Equipment

**Figure 5:**
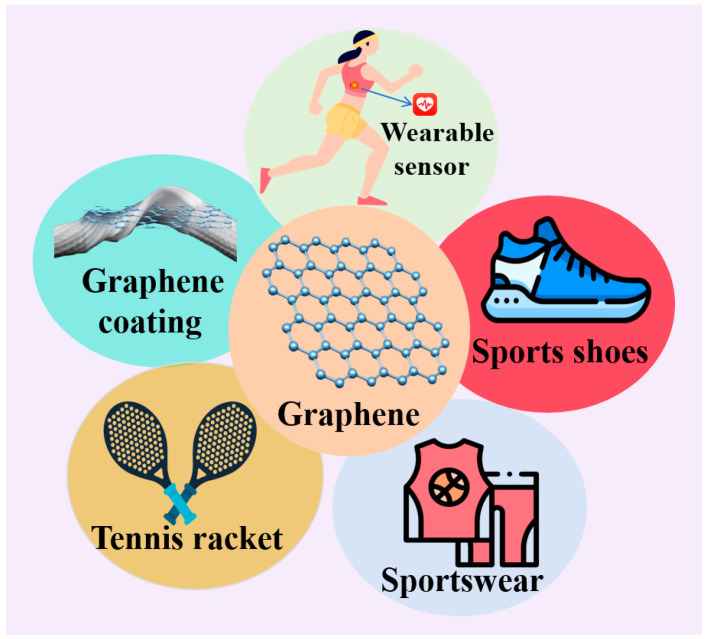
Graphene applications in the sports industry.

In today’s rapidly advancing society of science and technology, as we address the contradiction between pushing the limits of human athletic abilities and sports training, the performance of sports equipment consistently plays a crucial role in influencing athletes’ performance. To a certain extent, it enhances the ability of the human body to engage in physical activities. According to the specific requirements of different sports events, high-performance sports equipment has unique advantages in material selection, process design, and ergonomics. They also play a significant auxiliary role in improving athletes’ experience, performance, and safety. Figure 5 summarizes some applications of graphene in the sports industry.

### 5.1. Graphene Wearable Sensors

Scientific training in modern competitive sports relies on intelligent motion monitoring devices that provide athletes with physiological signals during training or competition [124]. Researchers have proposed various types of flexible electronic sensors. Graphene-based flexible wearable electrodes are considered efficient and promising because of their higher elasticity, flexibility, and hydrophobicity, which can better retain contact with human skin and remain stable after long-term use [125,126,127]. Graphene-coated highly conductive textile electrodes are, therefore, expected to replace the traditional metal-based electrodes in health monitoring devices such as electrocardiograms (ECG), electroencephalograms (EEG), and electromyograms (EMG) [128]. Shanthi et al. [129] successfully developed a highly flexible and washable sports undergarment using graphene-coated textile electrodes by mat curing technology. The study showed that due to the hydrophobic nature of graphene when the textile electrode was exposed to sweat in maximum contact with the skin, its impedance, and conductivity were not disturbed by human sweat. Therefore, this sportswear could monitor high-quality ECG and heart rate under different conditions, such as rest, walking, and running. Graphene-coated textile electrodes exhibit excellent washability and are more suitable for long-term use than metal (Ag/AgCl) ECG electrodes, which are less flexible and stretchable. However, due to the chemical inertness of the graphene surface and the absence of dangling bonds to effectively control the channel carriers, the dielectric layer should be thin by a few nanometers. Li et al. [130] achieved this by optimizing pre-H_2_O treatment and employing a two-step temperature growth atomic layer deposition technique. This way, they controlled the gas–solid physical adsorption between water molecules and the graphene surface, directly coating a uniform and dense Al_2_O_3_ film on graphene with a thickness of 5 nm. Consequently, the quality of the graphene–alumina was comparable to the optimal quality of the silicon–alumina film. Raza et al. [131] designed graphene textile (IGT) sensors by converting different polymer substrates into laser-induced graphene (LIG) and applying them to volleyball sportswear. The results showed that IGT sensors had four functions in volleyball sportswear: catch detection, finger contact foul detection when blocking the ball, spike force measurement, and player position monitoring. It is anticipated that graphene-wearable electronic sensors will improve the consistency between actual sports and virtual activities while enhancing the ability to correct movements in virtual space, thus providing unlimited opportunities for improving competitive levels.

### 5.2. Graphene Sneakers

Sports shoes can be divided into many types depending on the application requirements. Generally, polymer foams, especially ethylene vinyl acetates (EVA), are widely used as midsole cushioning materials in these articles. Therefore, the key to further improving the performance of sports shoes is to develop polymer foams with the required properties. Graphene is a carbon-based 2D material with potential advantages for improving polymer matrices’ mechanical, electrical, thermal, and electromagnetic wave absorption properties [132]. The molecular dynamics (MDs) modeling of the interaction between the graphene and EVA matrix showed that adding graphene increases Young’s modulus, the yield strength, and the glass transition temperature of the EVA matrix. When the solid interfacial bonding between the graphene additive and the EVA matrix (the graphene content is 9 wt.%) limits the mobility and flexibility of the EVA chain, the mechanical strength and thermal stability of EVA are improved [133]. Lunchev et al. [134] mixed graphene contents in EVA foam with a twin-screw extruder. After mechanical property analysis, graphene/EVA foam sports shoes were prepared with a sample containing 0.2 phr graphene. The results indicated that the graphene/EVA foam sneakers had a 40% increase in wear resistance and a 30% increase in axial stiffness compared to the pure EVA foam reference sneakers. This increase in the mechanical properties could effectively improve propulsion during running. At the same time, the compression testing revealed that the graphene/EVA foam sneakers were more flexible, which could help enhance their appearance and service life.

### 5.3. Graphene Tennis Racket

The design of tennis rackets constantly seeks to provide athletes with competitiveness, durability, and comfort; therefore, manufacturers are continually working to improve the materials and designs of their products. Graphene/polymer composites are graphene-dispersed in epoxy resin [135] and have higher strength and elastic modulus than ordinary materials. These composite materials can significantly improve the tennis rackets’ stiffness and strength. Moreover, graphene/polymer composites are generally less dense than aluminum, have strength comparable to steel, and have a high elastic modulus and fatigue strength [136], making them more suitable for tennis rackets. In 2013, HEAD launched a new series of tennis rackets (YouTek Graphene Speed series) made of graphene/polymer composites [137]. Young et al. [138] analyzed the microstructure of these rackets through combined optical microscopy and Raman spectroscopy. The study found that graphene-based nanoparticles were utilized in the heads of the tennis rackets and the areas where they connected to the handles, aiming to enhance the mechanical performance of these regions. Thus, the excellent performance of graphene/polymer composites makes rackets lighter and stiffer and lays a solid foundation for athletes to achieve good results.

### 5.4. Graphene Sportswear

Sports clothing must have a cooling function to rapidly release heat and sweat from the body through different pathways, such as conduction, convection, evaporation, and radiation [139], especially when there is a significant temperature difference between the body and the surrounding environment or surfaces in direct contact [140]. Graphene has become popular in textile applications, especially in lightweight sportswear, due to its excellent properties, high thermal conductivity, mechanical strength, antistatic effect, UV protection, electrical conductivity, and ease of functionalization. It has been used as a conductive filler to increase heat transfer in sportswear textiles [141]. Brazilian sportswear manufacturer BiaBrazil launched sportswear printed with graphene ink that is claimed to improve moisture management and heat transfer by 18% [142].

In boxing, the wrong training method can easily cause physical injury. Therefore, many sports protective clothing use high-performance materials to reduce the negative impact of boxing training. Liu used the orthogonal test method [143] to study the effect of materials and knitting structure on boxing clothing performance. Based on the characteristics of boxing injury, the researcher(s) prepared seamless boxing clothing using a graphene/polymer composite fiber. These graphene-based composite boxing suits were more comfortable and robust than ordinary ones and had excellent impact resistance, protecting the athletes from potential injuries. Li et al. [144] developed a form of compression clothing for running crafted from biomass-derived graphene-modified nylon fibers. The findings of the study revealed that the graphene-modified nylon fibers demonstrated a tensile strength of 4.52 cN/dtex, boasting superior mechanical properties compared to conventional nylon fibers. Additionally, it exhibited an outstanding low-temperature far-infrared (FIR) performance, with the FIR raising its temperature by 2.3 °C/30 s. This feature can enhance blood circulation and improve bodily functions, thereby amplifying athletic performance.

### 5.5. Graphene Coating

Anterior cruciate ligament (ACL) rupture is a common joint ligament injury, especially in young athletic people aged 16 to 39 [145]. Due to the poor healing ability of ruptured ACLs and the need for injured people to restore their function in time, artificial ligaments are becoming increasingly popular. Graphene can potentially promote cell attachment, proliferation, and differentiation and has shown excellent results in various biological applications in vivo and in vitro. Graphene has been the focus of tissue repair applications due to its excellent mechanical properties. Studies have shown that graphene can promote the differentiation of human bone marrow mesenchymal stem cells into osteoblasts while promoting the differentiation of human neural stem cells into neurons [146,147]. Wang et al. [148] investigated the effect of graphene coating on the bioactivity of PET-based artificial ligaments (PET-ALS). The results showed that bone tunnel healing rates were significantly accelerated after graphene-coated grafts were implanted in bone tunnels. Therefore, graphene-coated grafts can promote early recovery of ACL reconstruction and show great potential in enhancing the biological activity of the material surface.

### 5.6. Challenges of Biomass Waste Graphene in Sports Applications

Three-dimensional nanostructured graphene not only inherits some unique properties of two-dimensional graphene but also offers numerous novel features, such as a boosted surface area, rapid gas diffusion, and abundant reaction sites [149,150,151]. Its applications in the sports field are steadily expanding, encompassing enhancements in sports equipment performance and monitoring athletes’ physiological indicators. However, certain biomass wastes contain metal ions such as potassium and sodium, which can complicate the exfoliation process when deriving biomass graphene [152]. At the same time, varying temperatures and lignocellulose compositions can induce defects in the biowaste product composition, degradation rate, and other defects. Consequently, ensuring stability in the properties of the biowaste precursor materials becomes a key challenge [153]. In addition, heterogeneous waste precursor materials often contain small amounts of organic or inorganic impurities, necessitating suitable pretreatment [154]. Therefore, at an industrial scale, the costs of pretreatment and the requirements for supply chain stability may impede the large-scale production of biomass waste-derived graphene in the sports sector. 

However, although graphene derived from biomass waste has not yet been produced on an industrial scale in the sports sector, its conversion to graphene offers potential benefits over traditional methods. These include reduced emissions of exhaust gases, wastewater, and solid waste to some extent, as well as decreased energy consumption [155], which further promote the development of the green sports industry. 

## 6. Conclusions

This paper discusses the properties, production methods, and different biomass waste sources of graphene. Graphene has excellent electrical conductivity, biocompatibility, and mechanical properties compared to traditional carbon materials. Its application advantages in sports are mainly reflected in manufacturing lightweight and robust sports equipment and improving athletes’ comfort. At the same time, it has the potential to study the physiological mechanism of athletes deeply and promote training and rehabilitation. In addition, graphene is expected to shine in ice and snow sports based on its excellent thermal conductivity. Although graphene prepared from biomass waste is similar to graphene from traditional sources, the quality of the graphene produced is compromised due to most of the biological waste being heterogeneous in origin, thus limiting its large-scale application. Future research should focus on the sustainability and scalability of graphene production from natural resources. Graphene and its derivatives with superior performance can be produced on a large scale by improving the processing techniques and standardizing the sources and characteristics of biomass waste to meet the demands of various applications, including sports equipment manufacturing. Although graphene based on biomass waste is of poor quality, it still shows great potential for various applications.

## Figures and Tables

**Table 1:** Mechanical properties of the graphene family.

Materials	Experimental Technique	Young’s Modulus	Tensile Strength	Refs.
Monolayer graphene	Nano-indentation in AFM	1 TPa	130 GPa	[41]
Free standing GO	Nano-indentation on a dynamic contact tool	697 ± 15 GPa	3–33 GPa	[41]
RGO paper	-	41.8 GPa	293.3 MPa	[42]

**Table 2:** Comparison of different preparation methods for graphene.

Method	Substrate	Temperature (°C)	Yield	Refs.
Micromechanical exfoliation	SiO_2_/Si	Room temperature	Difficult to generate production output	[62]
Chemical vapor deposition	Cu, Pt, Ni, Ru, Ir	>1000	Can be produced on a large scale	[63]
Oxidation reduction	-	<500	Can be produced on a large scale	[64]
Epitaxial growth	SiC	1200–1600	Suitable for small-scale production	[65]
